# Infectious keratitis: A review

**DOI:** 10.1111/ceo.14113

**Published:** 2022-06-03

**Authors:** Maria Cabrera‐Aguas, Pauline Khoo, Stephanie L. Watson

**Affiliations:** ^1^ Save Sight Institute, Discipline of Ophthalmology, Faculty of Medicine and Health The University of Sydney Sydney New South Wales Australia; ^2^ Corneal Unit Sydney Eye Hospital Sydney New South Wales Australia

**Keywords:** acanthamoeba keratitis, bacterial keratitis, fungal keratitis, infectious keratitis, viral keratitis

## Abstract

Globally, infectious keratitis is the fifth leading cause of blindness. The main predisposing factors include contact lens wear, ocular injury and ocular surface disease. *Staphylococcus species, Pseudomonas aeruginosa, Fusarium species, Candida species* and *Acanthamoeba species* are the most common causal organisms. Culture of corneal scrapes is the preferred initial test to identify the culprit organism. Polymerase chain reaction (PCR) tests and in vivo confocal microscopy can complement the diagnosis. Empiric therapy is typically commenced with fluoroquinolones, or fortified antibiotics for bacterial keratitis; topical natamycin for fungal keratitis; and polyhexamethylene biguanide or chlorhexidine for acanthamoeba keratitis. Herpes simplex keratitis is mainly diagnosed clinically; however, PCR can also be used to confirm the initial diagnosis and in atypical cases. Antivirals and topical corticosteroids are indicated depending on the corneal layer infected. Vision impairment, blindness and even loss of the eye can occur with a delay in diagnosis and inappropriate antimicrobial therapy.

## 1 INTRODUCTION

Infectious keratitis is an infection of the cornea also known as infectious corneal ulcer or corneal opacity. Infectious keratitis can be classified as microbial keratitis (bacteria, fungi or parasites), or viral keratitis (herpes viruses).^1^, ^2^ The number of cases of corneal blindness due to infectious keratitis has decreased from about 1.6 million in 1990 to 1.3 million in 2015,^3^, ^4^, ^5^, ^6^ and of vision impairment from 3.3 million to 2.9 million cases during the same period,^4^ despite these data being underreported. Infectious keratitis is the most common cause of non‐trachomatous corneal opacification and the fifth leading cause of blindness overall causing 3.5% (36 million) of all blind individuals up to 2015.^5^ Epidemiological data for infectious keratitis is difficult to capture as most data are reported under ‘corneal blindness’ comprising traumatic, infectious, inflammatory and inherited conditions.^2^

Microbial keratitis incidence differs worldwide. In developed countries, the incidence has been reported at 27.6 per 100 000 years in the United States (US) in 1999, 40.3 per 100 000 in England in 2006, and 6.6 per 100 000 in Australia in 2015.^2^, ^7^ A contrasting situation is found in developing countries in Asia where infectious keratitis is a public health threat. These countries face difficulties in accessing health care, poor health indices and higher proportion of workers in farming and agriculture with incidences as high as 113 per 100 000 in Madurai, Tamil Nadu, India; 339 per 100 000 in Bhutan; 710 in Burma; and 799 in Nepal.^2^, ^3^, ^6^

In the United States, infectious keratitis is responsible for about 1 million visits to health professionals and 58 000 to emergency departments annually costing the US health system 175 million dollars in direct health expenditure.^1^, ^2^ The real burden of the disease worldwide is difficult to ascertain; however, poor rural and agricultural populations are likely to be disproportionately affected.^2^

## 2 BACTERIAL KERATITIS

Bacterial keratitis (BK) is the most common cause of microbial keratitis.^1^ It is an ophthalmic emergency requiring immediate attention as it can progress rapidly.^8^, ^9^ BK is one of the most common causes of visual impairment in working age adults.^3^

### 2.1 Predisposing factors

**FIGURE 1:**
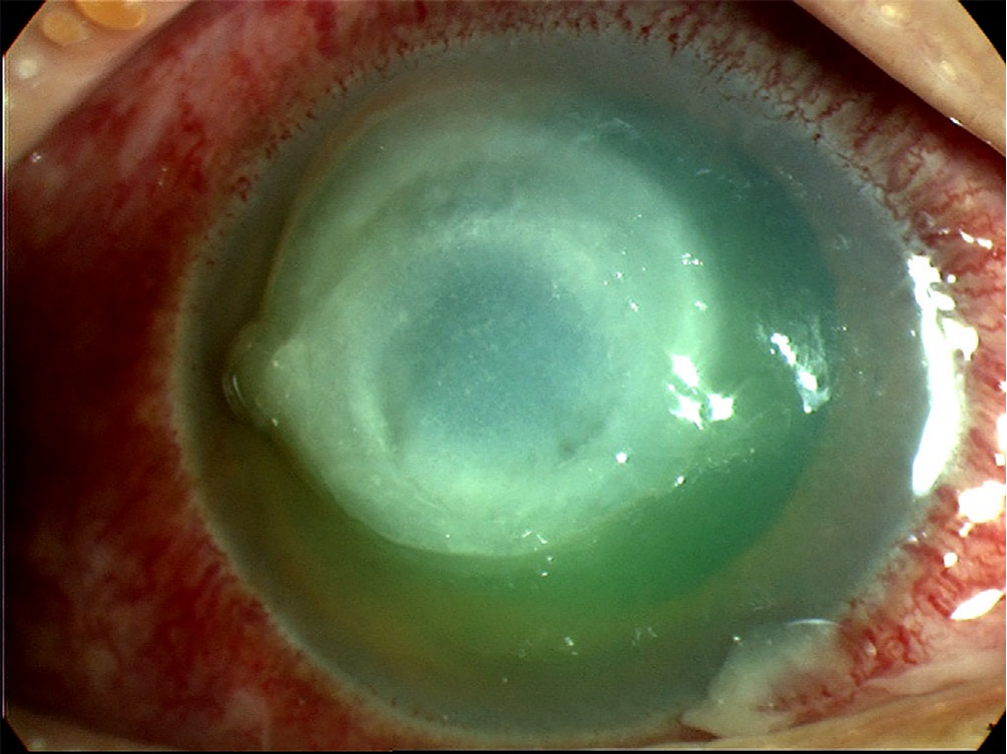
Slit lamp image of a case of bacterial keratitis in a contact lens wearer with typical features; there is a central corneal infiltrate with an overlying epithelial defect and conjunctival hyperaemia

**FIGURE 2:**
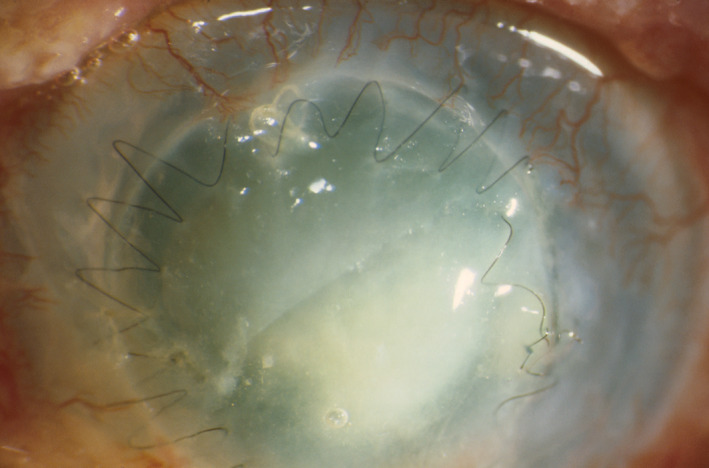
Bacterial keratitis in a failed corneal graft with a broken suture. The graft is oedematous and inferiorly a white infiltrate and larger epithelial defect can be seen within the graft. There is peripheral host vascularisation and conjunctival hyperaemia

**FIGURE 3:**
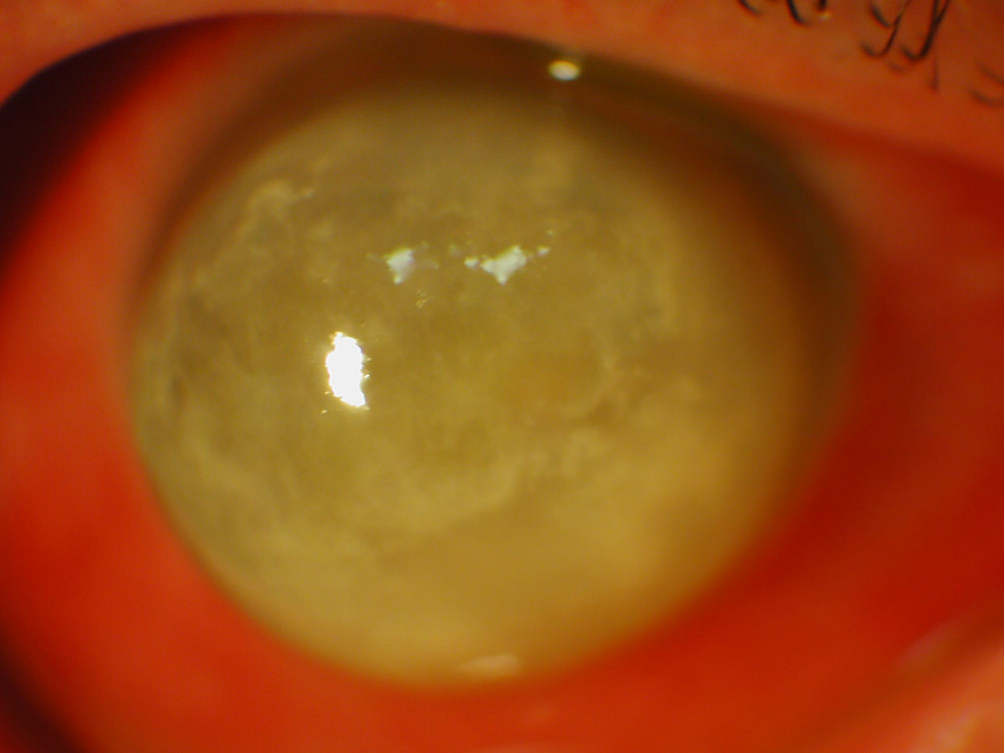
Slit lamp image of a protruding cornea with bacterial keratitis. The patient has keratoconus complicated by corneal hydrops and then bacterial infection. Scattered infiltrates can be seen across most of the protuberant cornea and the conjunctiva is hyperaemic

Predisposing risk factors for BK include contact lens wear (CLW, Figure 1)), previous topical steroid use, ocular surface disease (OSD), ocular trauma, previous keratitis and prior surgery (Figure 2) and corneal disease (Figure 3).^1^, ^10^, ^11^, ^12^ Studies have shown the risk of contact lens (CL)‐related keratitis decreases with age.^10^, ^13^ While the risk of keratitis related to a history of corneal transplant, previous ocular surgery within the last 3 months prior to infection, OSD and diabetes mellitus significantly increases with age.^10^, ^13^

The main risk factor for BK in developed countries is CLW^10^, ^14^ whereas trauma is the main risk factor in developing countries.^14^ In the United States, there are about 45 million contact lens wearers. In 2010, the estimated incidence of microbial keratitis cases per 100 000 person‐year was 130 among CLW versus 14 non‐wearers in the United States.^1^

### 2.2 Clinical features

Clinical features and symptoms of BK are described in Table 1. BK can occur in a range of clinical scenarios and present with variable clinical findings though most cases will have a corneal infiltrate, epithelial defect and conjunctival hyperemia as shown in Figures 1, 2, 3. Figure 1 illustrates a case of BK in a contact lens wearer with a central infiltrate and defect. In Figure 2, a failed corneal graft with a broken suture predisposed the cornea to infection and the signs of graft failure and BK are both present. Keratoconus can be complicated by corneal hydrops in which a break in Descemet's membrane produces corneal oedema, in Figure 3 the diffuse corneal oedema and BK have resulted in widespread corneal opacity and infiltrates.

**TABLE 1:** Summary of causal organism(s), clinical features, diagnostic tests and treatment of four types of infectious keratitis

Disease	Common pathogen	Clinical features	Transmission	Diagnostic tests	Treatment
Bacterial keratitis	*CoNS* *Staphylococcus aureus* *Streptococcus pneumoniae* *Pseudomonasaeruginosa*	**Symptoms**: Blurred vision, redness, photophobia. **Signs**: hypopyon, *Gram‐positive cocci*: localised round or oval corneal ulceration, greyish/white stromal infiltrates, distinct borders, minimal surrounding stromal haze. *Gram‐negative bacilli*: rapid, inflammatory course, dense stromal suppuration, hazy surrounding cornea, immune ring	Exposure to pathogens: CL wear Ocular surface disease Ocular trauma Topical steroid use Previous microbial keratitis	**Gram staining**: Sensitivity:60%–75% **Culture**: Sensitivity:38%–66% **PCR**: Sensitivity:25%–88%	Broad‐spectrum topical antibiotics^15^ Monotherapy with fluoroquinolones^a^ OR Fortified antibiotics: Cephazolin 5% plus gentamicin 0.9% Consider adjuvant topical steroid at least 2–3 days of improvement when: Organism has been identified and corneal infiltrate compromises the visual axis
Herpes simplex keratitis	Herpes simplex virus type 1	**Symptoms**: redness, discharge, watery eyes, irritations, itching, pain and photophobia **Epithelial HSK**: Dendritic or geographic ulcer **Stromal HSK**: Stromal haze/opacity without ulceration, lipid keratopathy, oedema, scarring, sectoral of diffuse neovascularisation, corneal thinning, immune ring **Endothelial HSK**: Central focal circular area stromal oedema, keratic precipitates **Keratouveitis**: Keratic precipitates, stromal keratitis, anterior chamber cells	Direct contact with infected lesions or their secretions.	**PCR**: Sensitivity: 70%–100% Specificity: 67.9%–98%	Australian HSK recommendations^16^: **Epithelial HSK**: Occ ACV 3% five times daily for 1–2 weeks OR VLC 500 mg BD, 7 days^b^ **Stromal HSK without ulceration**: VLC 500 mg once daily during topical steroid use PLUS Prednefrin Forte 4–6 times daily tapered over >10 weeks **Stromal HSK with ulceration**: VLC 1 g TDS for 7–10 days^c^ PLUS Prednefrin Forte twice daily tapered slowly as disease comes under control **Endothelial HSK**: VLC 500 mg once daily to 1 g TDS for 7–10 days^c^ (There is a lack of clinical evidence to guide dosage in this situation) PLUS Prednefrin Forte 4–6 times daily tapered over >10 weeks **Keratouveitis** ^d^: VLC 1 g TDS for 7–10 days^c^ PLUS Prednefrin Forte 4–6 times daily tapered over >10 weeks **Prophylaxis** ^e^: ACV 400 mg BD OR VLC 500 mg once daily
**Fungal keratitis**	**Filamentous fungi**: *Fusarium spp*. and *Aspergillus spp*. **Yeasts**: *Candida spp*.	**Symptoms**: Redness, tearing, pain, sensitivity to light, discharge, decreased vision **Signs**: *Filamentary fungi*: dry elevated slough, stromal infiltrate with hyphate margins, satellite lesions and thick endothelial exudate *Candida spp*.: stromal keratitis similar to bacterial keratitis, overlying epithelial defect, discrete infiltrate, slow progression, inferocentrally location	**Filamentous fungi**: Corneal injury Contact lens wear **Yeasts**: Ocular surface disease conditions: dry eye, blepharitis, bullous keratopathy, Steven‐Johnson Syndrome, exposure keratopathy	**Staining**: *Gram and Giemsa*: Sensitivity: 65%–75% 10% KOH: Sensitivity: 61%–99.23% Specificity: 91%–97%. **Culture**: Blood and chocolate and Sabouraud dextrose agar **PCR**: Sensitivity: 75%–100% Specificity: 50%–100% **IVCM**: Sensitivity: 80%–94% Specificity: 78%–91.1%.	**Filamentous fungi**: Topical natamycin 5% ***Candida spp***.: Topical voriconazole 1% Amphotericin B 0.15%
Acanthamoeba keratitis	*Acanthamoeba spp*.	**Signs**: Severe pain, tearing, discharge, decreased vision, FBS, photophobia, **Signs**: Corneal ulceration with ring‐shaped infiltrates, hypopyon, satellite lesions, conjunctival hyphemia, keratoneuritis or radial nerve enlargement with perineural infiltrates	Exposure to pathogens via CLs or trauma from contaminated soil or water.	**Culture**: Sensitivity: 31%–33% **PCR**: Sensitivity: 67%–75%	PHMB 0.02% Chlorhexidine 0.02%

Abbreviations: ACV, aciclovir; BD, twice daily; CL, contact lenses; CoNS, coagulase‐negative staphylococci; FBS, foreign body sensation; HSK, herpes simplex keratitis; IVCM, in vivo confocal microscopy; Occ, ointment; PCR, polymerase chain reaction; PHMB, polyhexamethylene biguanide; *spp*., species; TDS, three times daily; VLC, valaciclovir.

^a^ Ofloxacin 0.3%, ciprofloxacin 0.3%, moxifloxacin 5 mg/ml or levofloxacin 15 mg/ml depending on local availability and surveillance data.

^b^ Indications: immunocompromised patients; non‐compliance, inability to use or tolerate, or ocular toxicity from topical acyclovir.

^c^ Reduce valacyclovir to prophylactic dose after 7–10 days and maintain for as long as frequent topical steroids are in use.

^d^ Refer patient to cornea/uveitis clinic, respectively depending on degree of corneal or uveal involvement.

^e^ Indications: multiple recurrences of any type of HSK, especially stromal HSK; and patients with history of ocular HSV following any ocular surgery including penetrating keratoplasty or during immunosuppressive treatment.

The severity of BK can be divided into mild, moderate and severe. Mild corneal ulcers are those <2 mm in size with the depth of the ulcer <20% or 100 μm corneal thickness. Superficial infiltrates near the ulcer may also be seen. Moderate corneal ulcers range between 2 and 5 mm in size, depth of 20%–50% (100–275 μm) of the cornea, with dense infiltrates extending to the mid stroma. Severe ulcers are ≥5 mm, with a depth of more than 50% (>275 μm) and dense infiltrates reaching the deep layers of the corneal stroma.^17^ Poor patient outcomes have been associated with increased severity.^10^, ^17^

### 2.3 Diagnostic tests

A diagnosis of BK is made from the patient's history as well as microbiology tests. Preferably, all corneal ulcers should be cultured for the identification of the causal organism and the antibiotic susceptibility before commencing antimicrobial therapy.^11^ The American Academy of Ophthalmology (AAO), BK preferred practice pattern, recommends smears and/or cultures in the following situations^12^:

- Central and large corneal infiltrate and/or associated with significant stromal involvement or melting
- Chronic or unresponsive infection to broad‐spectrum antibiotic therapy
- History of corneal surgeries
- Atypical clinical features suggesting fungal, amoebic or mycobacterial keratitis
- Multiple infiltrates on the cornea

#### 2.3.1 Microbiology evaluation

Microbiology evaluation includes smear examination and culture of corneal scrapings into several media to grow organisms for identification.^18^ The culture media (two blood agars, chocolate agar, Sabouraud's agar slope and cooked meat medium) should be taken from the fridge and left for 1 h to reach room temperature. The corneal ulcer samples are then collected from the area of corneal infiltration using blades or typically 25‐gauge needles after instilling an anaesthetic eye drop (i.e., lignocaine 1%), with the first samples placed on glass slides for staining and then onto the media for culture.^14^, ^19^ Superficial corneal samples can be processed to 10% KOH‐calcoflour white wet mount, Gram or Giemsa staining onto glass slides for microscopy.^18^ Gram staining is beneficial providing prompt results in 5 min, can identify aerobic and anaerobic bacteria, fungi, amoeba and microsporidia, documents morphology of rods and cocci and distinguishes Gram‐positive and Gram‐negative organisms.^11^ Gram staining detects the type of organism in 60%–75% of bacterial cases.^14^

The positive culture rate from corneal scrapes ranges from 38% to 66%^6^, ^7^, ^9^, ^20^, ^21^, ^22^, ^23^, ^24^ from different studies worldwide.^25^, ^26^ In cases of progressive BK or where a negative result has been obtained from corneal scrapes or the organism identified does not match the clinical picture, a corneal biopsy can be performed. A lamellar corneal biopsy can be taken using a dermal trephine or freehand dissection, the specimen is divided into two halves to allow histopathological and microbiological analysis.^12^, ^27^

#### 2.3.2 Polymerase chain reaction test

There is a need for more sensitive and fast‐processing diagnostic methods due to the delay in identifying the causal organism(s) from corneal scrape cultures. Another test used in the diagnosis of microbial keratitis is the polymerase chain reaction (PCR) test.^28^ This is a molecular technique for the detection and analysis of specific DNA sequencies consisting of repeated cycles of denaturation, amplification and replication in which segments of DNA are continuously multiplied to enable their detection.^25^, ^28^, ^29^ All bacteria have the 16S ribosomal DNA (16S RNA) gene which consists in highly conserved regions of nucleotide sequences, interspersed with nine variable regions that are genus or species specific. The broad PCR primers target the conserved regions amplifying the variable regions. The genus or species of bacteria is identified after following sequencing and comparison to stored sequences in a database.^26^, ^30^ The advantages of PCR include its speed, sensitivity and cost‐effectiveness relative to culture and staining, ability to quickly differentiate bacterial and fungal ulcers, and the detection of slow‐growing bacteria and organisms that are traditionally difficult to cultivate or identify with traditional microbiological methods.^25^, ^26^, ^29^ On the other hand, the disadvantages include the high rate of false positive errors from commensal contaminants or dead bacteria, lower specificity compared with culture and staining, need to narrow the list of causative agents to use specific primers, difficulty for treating clinician to interpret which of the identified organisms is the causal one, less cost‐effective when performed with a multi‐organisms PCR approach, supply costs, equipment fees and training expenses.^25^, ^26^, ^29^

Different studies have compared culture versus PCR results in BK. Eleinen et al, reported the sensitivity to culture of 57.58% versus PCR sensitivity of 87.88%,^29^, ^31^ while Kim et al. reported a similar result for sensitivity to culture (56%) but lower PCR sensitivity (76%).^29^, ^32^ A study from Liverpool, United Kingdom (UK) reported that the overall BK detection rate was 36%, using culture and PCR analysis. Of these, 72.2% of isolates were detected by 16S rRNA gene PCR and 63.9% by culture. A combination of both PCR and culture detection methods significantly increased the overall isolation rate by 13% compared with using culture alone. Nevertheless, in negative cultures, 16S PCR yielded more results suggestive of potential organisms than cultures in 16S PCR negative samples, hence there were more 16S PCR positive samples with inconclusive results compared to cultures.^26^ Surprisingly, another study from the UK, reported bacterial PCR sensitivity of 25% versus culture of 95.6%. The authors discussed that the higher rates of PCR sensitivity in other studies may have been due to the detection of non‐pathogenic bacteria or better culture in combination with less effective PCR in their laboratories.^28^

The PCR related technologies also have an important role in diagnosing rare organisms such as atypical mycobacteria and *Nocardia species*.^33^ Atypical mycobacteria can be identified by a rapid and sensitive test such as the LightCycle system which combines real‐time PCR with fluorescence resonance energy transfer to obtain fast PCR results to identify different organisms.^23^, ^34^, ^35^ This system performs a melting curve analysis to differentiate closely related organisms including polyomaviruses, *Bordetella species* and *Bartonella species*.^34^, ^36^ Molecular tests such as PCR and gene sequencing with restriction endonuclease analysis of 16S rRNA gene and restriction fragment length polymorphism analysis of heat shock protein gene, DNA sequencing and pyrosequencing can be used for the identification of *Nocardia species*.^35^, ^37^, ^38^ Gene sequencing have identified several *Nocardia species* with a sensitivity of 88% and specificity of 76%. The PCR based hsp65 gene sequencing can isolate species causing ocular Nocardiosis.^38^ PCR has the advantage of detecting even fastidious microorganisms from a small specimen and can be rapidly performed compared with prolonged culture times for such organisms. However, PCR is expensive and not readily available at all sites.^33^, ^37^, ^38^ Despite these drawbacks, the evidence suggests that having multiple diagnostic tests available is needed to optimise the yield of positive cases to assist in an adequate diagnosis and antibiotic therapy.^28^

### 2.4 Microbiological patterns

The type of causative organism varies according to the patient's predisposing risk factors and geographical regions. However, despite local and regional variations in BK, the most commonly reported causative organisms appear consistent worldwide, with a higher proportion of infections caused by Gram‐positive (48%–89%) than Gram‐negative isolates (11%–50%).^2^ Caution is needed when interpreting results as most eyelid and ocular surface commensal organisms are Gram‐positive and likely to contaminate the sample.^39^ Nonetheless, the most common Gram‐positive organisms include *Staphylococcus aureus*, *Coagulase‐negative staphylococci* (CoNS), and *Streptococcus pneumoniae*.^1^, ^2^, ^9^, ^39^ Among Gram‐negative organisms, *Pseudomonas aeruginosa* has been reported to be the most common causative organism and has been implicated in BK among CLWs.^1^, ^10^ While, CoNS have been implicated in OSD patients.^40^

### 2.5 Treatment

#### 2.5.1 Antibiotic therapy

Adequate treatment for BK is key to avoid serious complications such as vision impairment or even the loss of the eye.^9^, ^12^ The initial treatment is generally empiric as culture results can take over 48 h, and the infection can progress rapidly without treatment. The mainstay of treatment is broad‐spectrum topical antibiotics which should be used until culture results are available (Table 1). Ocular ointment may be useful at bedtime in less severe cases or as adjunctive therapy. Subconjunctival antibiotics may be useful in scleral or intraocular infections.^12^ For central or severe keratitis, an initial frequent dosage every 5–15 min is recommended followed by hourly applications. Cycloplegic agents may be also used to decreased synechiae formation and reduce eye pain. They are indicated in cases with significant anterior chamber inflammation.^12^

The AAO BK Preferred Practice Pattern, the Royal College of Ophthalmologists Focus, UK and the Australian Therapeutics Guidelines initially recommend monotherapy with fluoroquinolones (ciprofloxacin 3 mg/ml, ofloxacin 3 mg/ml, moxifloxacin 5 mg/ml, levofloxacin 15 mg/ml, gatifloxacin 3 mg/ml or besifloxacin 6 mg/ml). An alternative includes a combination of cephalosporin or vancomycin plus and an aminoglycoside. Vancomycin should be used in case of multi‐drug resistant Gram‐positive isolates^11^, ^12^, ^15^, ^41^ The current guidelines in Australia recommend empiric therapy with fluoroquinolones; 0.3% ciprofloxacin or 0.3% ofloxacin, or fortified combination therapy with 5% cephazolin plus 0.9% gentamicin; either treatment with one drop every hour including overnight.^15^

Treatment should be modified based on the results of culture and susceptibility testing.^12^ In patients with a history of OSD, care should be taken when prescribing fortified antibiotics to these patients, as fortified antibiotics have been reported to have drug toxicity five times greater than ofloxacin alone. Furthermore, poorer patient outcomes have been reported in OSD patients who were prescribed combination fortified antibiotics when compared with ofloxacin alone.^40^

#### 2.5.2 Antimicrobial resistance

Generally, BK cases respond to either of the above therapies; however, increasing resistance to fluoroquinolones has been reported in the US since the 1990s.^9^, ^14^, ^42^ Goldstein et al. reported an increasing trend in resistance for ciprofloxacin in *S. aureus* (5.8% to 35%) and CoNS (15% to 39%) cases and a significant resistance among *Streptococcus species* (50%) during 1993 and 1997 in Pittsburg.^42^ The Antibiotic Resistance Monitoring in Ocular Microorganisms (ARMOUR) cumulative report from 2009 to 2018 reported that 34.9% of *S. aureus* were methicillin‐resistant *S. aureus* (MRSA). Resistance to ciprofloxacin was 32.2% among all *S. aureus* (10.4% for Methicillin‐sensitive *S. aureus* and 72.7% for MRSA) and 32.2% for CoNS.^43^ In addition, the ARMOUR cumulative report from 2009 to 2020 reported a decreasing trend in resistance noted to ciprofloxacin among *S. aureus* (39% to 33%) and CoNS (46% to 26%).^44^ On the other hand, in Australia, the Bacterial Ocular Surveillance System reported lower rates of resistance to ciprofloxacin with 16% among all isolates of *S. aureus* and 6% of CoNS.^8^, ^9^, ^14^, ^42^

#### 2.5.3 Topical corticosteroid therapy

The use of adjuvant topical corticosteroid therapy remains controversial.^14^, ^45^, ^46^ The aim of this therapy is the suppression of inflammation to reduce corneal scarring, neovascularisation and vision loss. However, the disadvantages include worsening of the infection, local immunosuppression, corneal melting and increased intraocular pressure.^12^, ^46^ The SCUT trial evaluated the effect of adjunctive corticosteroids (topical prednisolone phosphate, 1.0%) on clinical outcomes in patients with BK. At 12 months, the trial concluded that the adjunctive therapy may be associated with improved clinical outcomes in culture proven non‐*Nocardia* BK after at least 48 h of improvement with antibiotic therapy.^12^, ^41^, ^45^ If the corneal infiltrate compromises the visual axis, topical corticosteroid may be added to the management after at least 2–3 days of improvement with topical antibiotics, when the causal organism has been identified and it is not a fungus for which corticosteroids are contraindicated.^12^

### 2.6 Complications

Surgical interventions are indicated in severe lesions that present progressive stromal thinning, descemetocele formation and local perforation.^41^ The application of cyanoacrylate tissue adhesive is the first line intervention for corneal perforation providing a successful tectonic support for a short time, although requiring reapplication with a month after first application.^41^, ^47^, ^48^, ^49^, ^50^ The success of this adhesive ranges between 29% and 86% depending on the cause of the perforation, indications for applications and definition of success.^47^ Complications associated with its application include increased ocular inflammation, corneal neovascularisation and giant papillary conjunctivitis as well as long‐term adhesion.^41^, ^47^, ^48^, ^49^, ^50^ Another alternative is amniotic membrane transplantation which has anti‐inflammatory effects to accelerate corneal healing.^41^ However, a therapeutic penetrating keratoplasty (PK) remains the major intervention for the management of rapidly progressing infections and in large corneal perforations.^41^ Although it is usually a successful intervention, the probability of graft survival is reduced in about a half, at 4 years post‐intervention, in eyes with inflammation or with corticosteroid use at the time of graft.^41^, ^51^

### 2.7 Future direction in diagnosis of BK

#### 2.7.1 Metagenomics next‐generation sequencing

A promising diagnostic test is the metagenomics next‐generation sequencing (NGS). Ideally, NGS can detect all the microorganisms from a sample, producing sequencing data to be decoded potentially improving diagnostic yield, as it is inherently unbiased and hypothesis‐free.^30^ Targeted amplicon sequencing and metagenomics (mNGS) are two approaches to NGS. The first technique consists in primer‐mediated amplification of specific suspected genomic targets (16S rRNA for bacteria). Selective amplification and sequencing can also be used for probing genomic regions of special interest (loci that confer AMR). This approach is less expensive, provides more depth in complex microbial communities and has successfully studied genomes in molecular epidemiological studies of Zika and Ebola. In contrast, with a single primer set, the search for organisms across multiple microbial kingdoms is not feasible. For example, by sequencing only conserved genes such as 16S rRNA, low taxonomic resolution is provided with restrictions to the identification of organisms at genus level generating false‐positive results.^52^

Metagenomics NGS amplifies all nucleic acids within specimens without a target providing a considerable number of reads. Nevertheless, offering quantifiable phylogenetic identification of both known and unknown organisms within a specimen. This approach has been used as the last alternative to identify organisms in patients with severe systemic diseases when conventional tests have failed in identifying the causing organism. This approach also assists in molecular epidemiology studies investigating biogeographical and spatial distributions of pathogens in the context of their metagenome, and in high resolution evolutionary and outbreak tracing.^52^ Challenges include that the turnaround time is about 5–7 days similar to a standard culture, but with higher costs. Currently, if this approach yields a result not obtained in the culture, independent confirmation of this result with another assay in a certified laboratory is needed. With a culture sensitivity between 30% and 60%, this will occur frequently.^53^ Another challenge is how to determine whether a potentially contaminant organism is the actual causal organism of the infection. Perhaps other comparative sequence analysis algorithms may be needed to be explored.^11^, ^53^

Although NGS and dot matrix hybridization can simultaneously detect target pathogens or specific gene loci, NGS is not ideal for clinical use. NGS requires amplification of the target sequence or enrichment of desirable DNA sequences along with post‐sequencing analysis.

#### 2.7.2 Deep learning

Deep learning algorithms are increasingly being recognised as having potential for screening and making management recommendations for patients with painful red eyes^54^; distinguishing active corneal infection from scarring^55^; and differentiating between causal organisms in keratitis^56^—for example between fungal and BK.^57^ Convolutional neural networks apply very effectively deep learning for image classification. Algorithms such as ResNet, DenseNet, ResNeXt, SENet, VGG and EfcientNet can potentially develop models for image diagnosis of BK.^54^, ^57^ A study from Thailand used three algorithms, DenseNet121, REstNet50, VGG19 to classify images of patients with infectious keratitis. The test accuracy (F1 score) was higher for VGG19 (78%) followed by DenseNet121 (71%) and REstNet50 (68%). The authors created their own model called Deepkeratitis combining these algorithms with a F1 score of 83% which showed the best performance in differentiating BK from fungal keratitis (FK) compared with single models.^57^ Investigators using external eye photographs to assess deep learning frameworks in BK have reported that the diagnostic accuracy of different models ranged from 69% to 72%; comparable to ophthalmologists (66% to 74%).^54^ In areas or circumstances where patients are unable to access ophthalmic care, the ability to diagnose and assess microbial keratitis through artificial intelligence using external eye photos, such as could be taken with a mobile phone, may allow for appropriate therapy to be commenced without delay.^2^, ^5^, ^54^, ^55^, ^56^

## 3 HERPES SIMPLEX KERATITIS

### 3.1 Epidemiology

Herpes simplex virus keratitis (HSK) is a leading cause of monocular infectious blindness in developed countries due to stromal opacification.^58^, ^59^, ^60^ Herpes simplex virus (HSV) is an enveloped double‐stranded DNA virus belonging to the Herpesviridae family responsible for this corneal infection. This virus has two forms: HSV‐1, more related to ocular and perioral disease and HSV‐2 with anogenital infections.^58^, ^61^, ^62^ In the United States, an estimated 500 000 people have ocular HSV infection which treatment of new and recurrent cases costs the country US$ 17.7 million annually.^61^, ^63^ One of five people with ocular HSV infection can develop stromal HSK with the attendant risk of blindness.^58^ In 2012, Farooq and Shukla, estimated the incidence of HSK at about 1.5 million, with 40 000 new cases of severe monocular visual impairment or blindness each year across the world.^61^ Herpes simplex keratitis needs frequent visits to the ophthalmologist and is responsible to loss of work and productivity, and income.^64^ In the US in 2003, it was estimated that the HSK treatment cost in excess of 17.7 million dollars annually representing an important burden to the healthcare system.^65^ HSK is a leading cause of monocular infectious blindness in developed countries due to stromal opacification.^58^, ^59^, ^60^

#### 3.1.1 Predisposing factors

The susceptibility of the host to the virus and the local susceptibility of the host target tissue determine the severity and frequency of recurrent HSK episodes. The susceptibility of the host to the virus is driven by their immune status; therefore, any inherited or acquired immunosuppressive conditions, age and atopy increase the frequency of HSK recurrences or severe disease.^65^ Some immunosuppressive conditions include organ transplant recipients, diabetes mellitus, measles infections and human immunodeficiency virus (HIV).^65^, ^66^, ^67^ In terms of age, as children generally have a more robust immune response, they tend to present with severe ocular HSV inflammatory disease, more recurrences, and complications compared to adults.^61^, ^65^, ^68^ Complications include stromal scarring, corneal opacification, irregular astigmatism and amblyopia.^65^ Children present more commonly with bilateral ocular HSV disease in primary infections than in recurrent infections, with rates ranging from 3.4% to 26% and a recurrence rate within the first year of 45 to 50%, when compared to adults (1.3% to 12% and 18%, respectively).^65^ In a study from California, the United States, patients with severe atopic disease had between 2 and 4.8‐fold higher odds to have ocular HSV disease than people without atopy.^61^, ^65^, ^67^, ^68^

The local susceptibility of the cornea may be affected in cases such as application of medications, trauma and inflammation.^65^ Medications such as prostaglandin agonists (latanoprost) for the management of elevated intraocular pressure and corticosteroids may increase the risk of recurrent ocular HSV disease.^65^ Any surgery on an eye with previous ocular HSV disease increases the risk of recurrence of the infection.^65^, ^69^ The trauma caused by the surgery and the local immunosuppression of the perioperative corticosteroids may contribute this recurrence. Hence, the recommendation of an antiviral prophylactic therapy in the immediate perioperative period especially while the patient is also on corticosteroid therapy.^65^, ^67^ The Australian Corneal Graft Registry reported that penetrating grafts with active HSV have a probability of survival of 0.58 versus grafts with history of HSV with survival of 0.83 at year 4 post‐graft.^51^, ^67^

#### 3.1.2 Clinical diagnosis

Primary HSV infection can be transmitted by direct contact with infected lesion or their secretions. It generally occurs upon exposure to virus shed asymptomatically by mucosal tissues with an incubation period from 1 to 28 days.^59^, ^64^ After primary infection, the HSV spreads via retrograde axonal transport to establish a latent infection in sensory nerve ganglia including the trigeminal ganglion. Recurrent infections occur when there is a viral reactivation transporting the virus down to the eye.^59^, ^64^, ^67^

**FIGURE 4:**
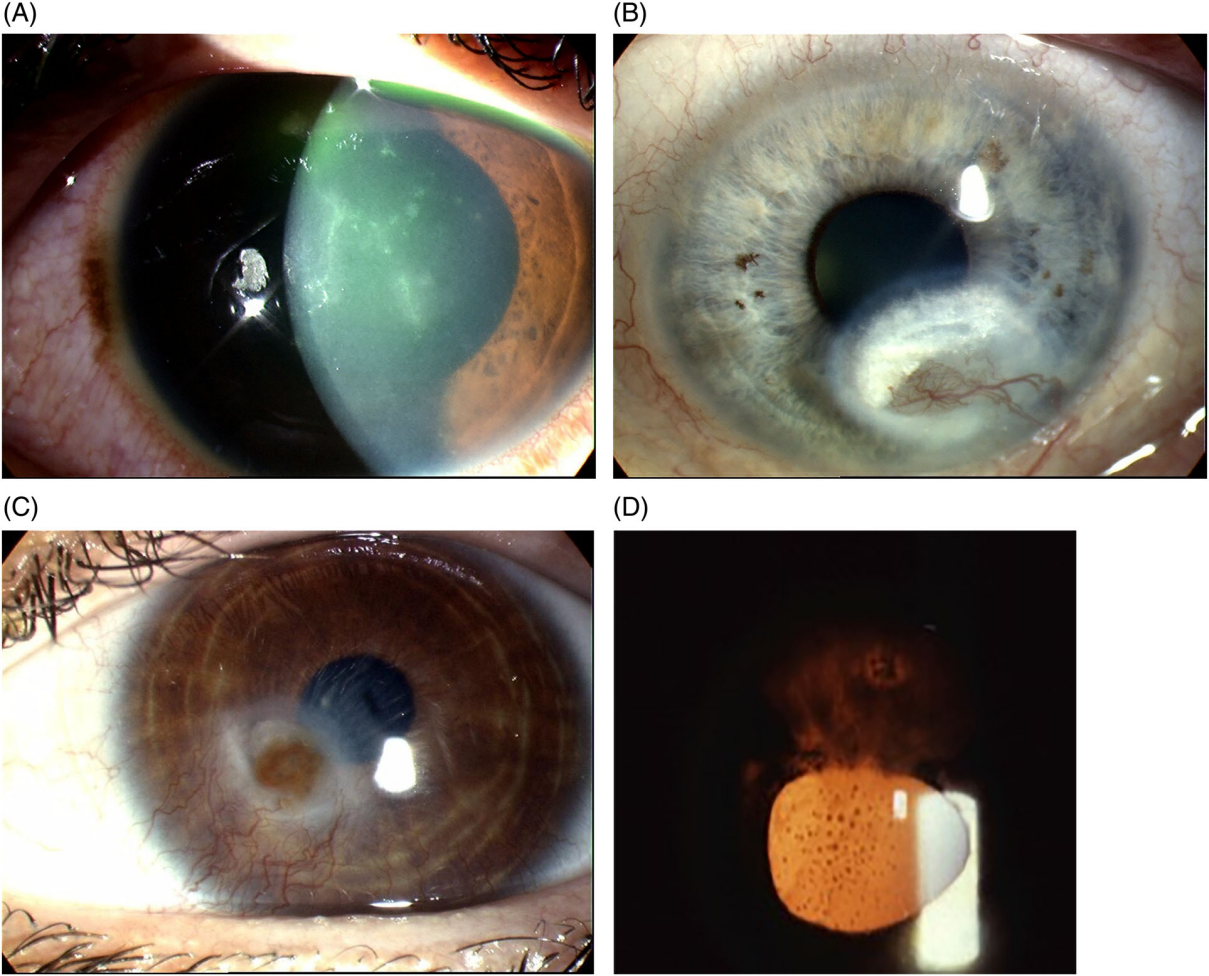
(A) Dendritic ulcer in epithelial herpes simplex keratitis stained with fluorescein. (B) Stromal herpes simplex keratitis with lipid keratopathy and vascularisation. (C) Stromal herpes simplex keratitis with ulceration. (D) Herpes simplex keratouveitis with anterior chamber cells

A diagnosis of HSK is made under clinical examination and after evaluating the patient's medical history. A history of labial cold sores or history of HSK could be the first clues to the diagnosis.^60^ The clinical features and signs vary with the type of HSK and chronicity of the disease as summarised in Table 1 and illustrated in Figure 4.^58^, ^59^, ^60^, ^64^, ^65^ A classification system based on the type of corneal layer infected was introduced in the ‘Herpes Simplex Virus Keratitis: a treatment guideline’ by the AAO in 2014.^65^ Epithelial HSK typically presents with a characteristic epithelial dendritic ulcer (Figure 4B). Whereas in stromal HSK, lipid keratopathy and vascularisation are classic features of chronic disease (Figure 4B) and ulceration may occur acutely (Figure 4C). In keratouveitis (Figure 4D) anterior chamber inflammation is associated with signs of HSK (Figure 4D).

#### 3.1.3 Diagnostic tests

Diagnostic tests maybe requested in the following cases: to confirm the initial diagnosis, atypical or complicated cases, uncertain diagnosis and suspected neonatal HSV infection. Viral culture is considered as the gold standard for epithelial HSK. It has a high specificity; yet with a limited use in clinical settings due to its low sensitivity, need of a skilled technician and slow turnaround (up to 10 days).^65^, ^66^

The PCR test detects viral DNA and quantifies the number of viral copies differentiating viral shedding from replication.^64^ In HSK, the specimen for PCR is typically obtained by swabbing an active herpetic lesion such as an epithelial keratitis or stromal keratitis with ulceration. Advantages of the PCR test include its high sensitivity and fast results. Disadvantages include the need for a skilled technician, special equipment and appropriate facilities with parameters for ocular samples, and inability to differentiate HSV shedding from infection.^64^, ^65^ Diverse studies have determined the sensitivity of PCR testing to be between 70% and 100% and specificity of 67.9% to 98%.^69^, ^70^, ^71^ However, in a retrospective case series from Sydney, Australia, the overall PCR positivity rate was 27%. It should be noted that 34% of epithelial HSK cases and 39% of stromal HSK with ulceration cases had a positive PCR contrasting to zero stromal HSK without ulceration cases and zero cases of endothelial HSK.^72^ This confirms that the interpretation of the PCR test is more likely to diagnose patients with typical lesions or patients who have not used antiviral medications.^63^, ^64^, ^72^ Nonetheless it can be a useful test when it is able to confirm for the clinician and patient that keratitis is due to HSV. As in cases of recurrent keratitis a diagnosis of HSK can then be readily made.

#### 3.1.4 Treatment

The appropriate therapy for each type of HSK generally depends on the correct diagnosis under clinical examination. The current treatment recommendations for HSK treatment were based on the results of Herpetic Eye Disease Study (HEDS) group clinical trials in the 1990s.^58^, ^63^, ^65^, ^67^ However, there are currently newer antivirals and the availability of them varies according to the country.^58^, ^65^, ^67^ The AAO released a treatment guideline in 2014 which recommended ganciclovir as the first line topical therapy with alternatives such as oral aciclovir, famciclovir and trifluridine for epithelial HSK.^65^, ^69^ However, ganciclovir and trifluridine are not easily accessible in Australia as they are in the United States, where topical aciclovir is not Food and Drug Administration (FDA) approved for HSK.^58^ A study from Sydney, Australia, found diverse prescribing patterns for HSK therapeutic and prophylactic treatments. These were not aligned to the HEDS treatment recommendations.^58^ As a result, an evidence‐based HSK treatment guideline was developed, implemented and evaluated to standardise the initial treatment for this condition (Table 1).^16^, ^58^, ^60^, ^61^, ^65^ The Royal Australian and New Zealand College of Ophthalmologists (RANZCO) endorsed the treatment guideline in April 2020.

#### 3.1.5 Complications

Recurrent HSK episodes can damage the corneal nerves causing neurotrophic keratopathy. Patients present with a decreased corneal sensation from irregular epithelial surface to an oval‐shaped neurotrophic ulcer with a heaped‐up border, blink reflex and tear production due to the damage to the sensory fibres innervating the cornea.^63^, ^69^ The infection causes a significant regression of the sensory afferents innervating the cornea, particularly substance P and calcitonin gene‐related protein nociceptive fibres with the loss of corneal sensitivity.^63^ Substance P and calcitonin gene‐related protein are neuropeptides involved in the epithelial renewal and wound repair. Following the infection, the cornea reinnervates but with a different organisation of its fibres and reduced concentrations of the substance P. If the breakdown of the epithelium is not appropriately treated early, it may lead to corneal scarring, thinning, vascularisation, perforation or secondary corneal infection.^63^

There are diverse treatments to stimulate epithelial growth and prevent further disruption of the ocular surface depending on the severity of the condition. For early and moderate cases, ocular lubricants, bandage contact lens, tarsorrhaphy, botulinum toxin‐induced ptosis, growth factors and autologous plasma maybe indicated. For more severe and complicated cases, collagenase inhibitors, tissue adhesives, conjunctival flap, amniotic membrane use and PK or lamellar keratoplasty can be used considering that poorer outcomes occur more in severely anaesthetic corneas.^63^, ^69^

### 3.2 Herpes zoster keratitis

Herpes zoster keratitis (HZK) usually manifests within 1 month of the onset of Herpes zoster ophthalmicus (HZO) and can affect any layer of the cornea. About 6% to 10% of cases of HZO can present with vision loss mainly due to corneal scarring or haze following acute epithelial and/or stromal HZK.^61^, ^74^, ^75^

#### 3.2.1 Epidemiology

It has been estimated that 200 000 new cases of HZO occur each year in the US.^76^, ^77^, ^78^ Varicella zoster virus (VZV) is highly prevalent in the general population, with rates between 97.5% and 100% for 5 to 9 and 75‐ to 79‐year‐olds.^79^ A trend towards younger age at presentation for HZO has been reported and maybe associated with childhood varicella vaccination.^77^, ^78^ Further, vaccination of older adults is increasing due to its effectiveness in reducing disease burden of HZO.^61^, ^80^, ^81^

#### 3.2.2 Predisposing factors

HZO occurs due to the reactivation of latent VZV from the ophthalmic division of the trigeminal nerve. Similar to HSK, primary infection follows latent and recurrent infection and is frequently associated with chronic and/or recurrent disease.^80^ Predisposing factors for HZO and HZK include immunosuppression, advancing age, overexposure to the sun, a family history, trauma and ocular surgery such as cataract surgery.^60^, ^81^, ^82^, ^83^, ^84^ Recently, COVID‐19 vaccination may predispose to HZO.^85^

#### 3.2.3 Clinical diagnosis

The clinical appearance of HZK depends on the layer of the cornea affected. Epithelial HZK is common and occurs in about half of patients with ocular involvement in HZO. In epithelial HZK, punctuate epithelial lesions appear 2 days after the onset of the vesicular skin rash. At around day 6, the epithelial lesions form pseudodendrites, which are small and fine lesions in a branching pattern, formed by swollen and heaped up corneal epithelial cells.^74^, ^82^, ^87^ In contrast to the dendritic ulcers in HSK, the pseudodendrites lack of terminal bulbs and are usually located more in the peripheral cornea. They generally resolve spontaneously, however, in around half of cases, there is a progression to stromal HZK.^74^

Stromal HZK presents in 6%–16% of patients with ocular involvement of HZO. Stromal HZK usually manifests after the epithelial disease and at around day 10 after the onset of HZO. Signs include stromal opacity, vascularisation, nummular corneal opacity, scarring and lipid keratopathy.^74^, ^82^ Keratouveitis/endotheliitis occurs rarely in up to 7% of patients within a week of the onset of HZO. Signs include localised corneal oedema, cell and flare and a complement‐mediated immune Wessely ring, elevated intraocular pressure, anterior chamber involvement, and hypopyon or hyphema from the vasculitis in severe cases.^74^, ^82^

#### 3.2.4 Diagnostic tests

The diagnosis of HZK is usually made clinically on examination. In the acute phase, vesicular lesions maybe seen on the forehead and chronically there maybe scarring in the ophthalmic division of the trigeminal nerve. A swab may be taken from a vesicular skin lesion, a corneal lesion or AC tap for a PCR test to detect VZV DNA with rapid and sensitive results.^88^ Higher VZV DNA copy numbers have been associated with more recurrent disease.^88^

#### 3.2.5 Treatment

Oral antiviral agents should be commenced within 72 h of onset of HZO; Aciclovir, valaciclovir and famciclovir can be used.^61^, ^74^, ^89^, ^90^, ^91^, ^92^ Despite treatment recommendations for patients with HZO, there is little consensus on the management of keratitis.^87^ Diverse antiviral agents alone or in combination with topical corticosteroids can be effective for pseudodendritic keratitis despite current or recent oral antiviral therapy.^93^, ^94^, ^95^ For instance, topical ganciclovir 0.15% gel was successful in these cases.^74^, ^87^ Topical corticosteroid use aims to control the inflammation in stromal and keratouveitis/endotheliitis cases; but may be challenging to taper off. Clinicians should monitor for side effects such as glaucoma and cataracts when topical corticosteroids are used.^74^

#### 3.2.6 Complications

In the long‐term stromal inflammation from HZV can result in stromal keratitis with corneal vascularization and lipid keratopathy, scarring and possible perforation. Nerve damage may lead to neurotrophic keratopathy with loss of corneal sensation and of corneal epithelial integrity and tear dysfunction.^82^ Neurotrophic keratopathy may manifest months after HZO with diffuse epitheliopathy and chronic surface dysfunction and followed by band keratopathy. Corneal oedema can occur as the chronic end stage of corneal endothelial destruction caused by the virus or the related inflammation in keratouveitis/endotheliitis cases.^82^ Corneal mucous plaques or delayed pseudodendrites may also occur months or year later typically in a quiescent eye.^61^

### 3.3 Fungal keratitis

FK is a devastating condition and one of the main causes of blindness in Asia.^97^, ^98^ FK accounts for 6% to 53% of all cases of infectious keratitis depending on the country.^97^, ^99^, ^100^ Predisposing factors, causal organisms and clinical outcomes depend on the geographic location, occupation, available medications, and gross national income.^98^

#### 3.3.1 Predisposing factors and microbiology

Corneal injury, microtrauma with CL wear, medical history of systemic conditions, topical corticosteroid use and history of OSD such as dry eye, blepharitis, Steven‐Johnson syndrome, bullous keratopathy and exposure keratitis and are the main predisposing factors.^98^, ^100^, ^101^ In a corneal injury with vegetative matter or objects contaminated with soil, the fungus is introduced directly into the epithelial defect or the defect is infected during the trauma. This type of trauma occurs mainly in individuals working in farms, agriculture or outdoor settings.^102^ Filamentary saprophytic fungi are more commonly associated with corneal injuries and are more prevalent in tropical and sub‐tropical climates.^103^, ^104^ A study from India reported that 90% of FK cases were caused by injury while 11%–44% of FK in the United States were injury related.

Hard and soft‐extended CLW are related to *P. aeruginosa* keratitis; but filamentous and yeasts have been also associated with CLW. For example, *Candida albicans* can adhere to CL secreting exopolymers almost impenetrable to antibiotics and difficult to remove. In addition, this type of contact lens causes relative hypoxia of the corneal epithelium which may modify the cell surface glycoproteins. Microtrauma due to CLW can increase the organism adherence to the non‐adherent epithelium. Fungi and bacteria adherent to CLs come from poor CL handling including cleaning and lenses storage.^102^ *Candida species* is more commonly found in temperate climates, in diverse environmental settings and is part of the normal human microbiome. It is commonly found as a commensal organism in human gut, respiratory and mucous membranes. Candida related FK is more common in patients with prior OSD, recent ocular surgery and topical immunosuppression.^98^, ^102^, ^103^, ^104^, ^105^ The most common filamentary fungi include *Fusarium species*, *Aspergillus species* and *Curvularia species* and the most common yeasts, *C. albicans* and *Candida parapsilosis*.^98^, ^99^, ^103^, ^105^, ^106^

#### 3.3.2 Clinical presentation

**FIGURE 5:**
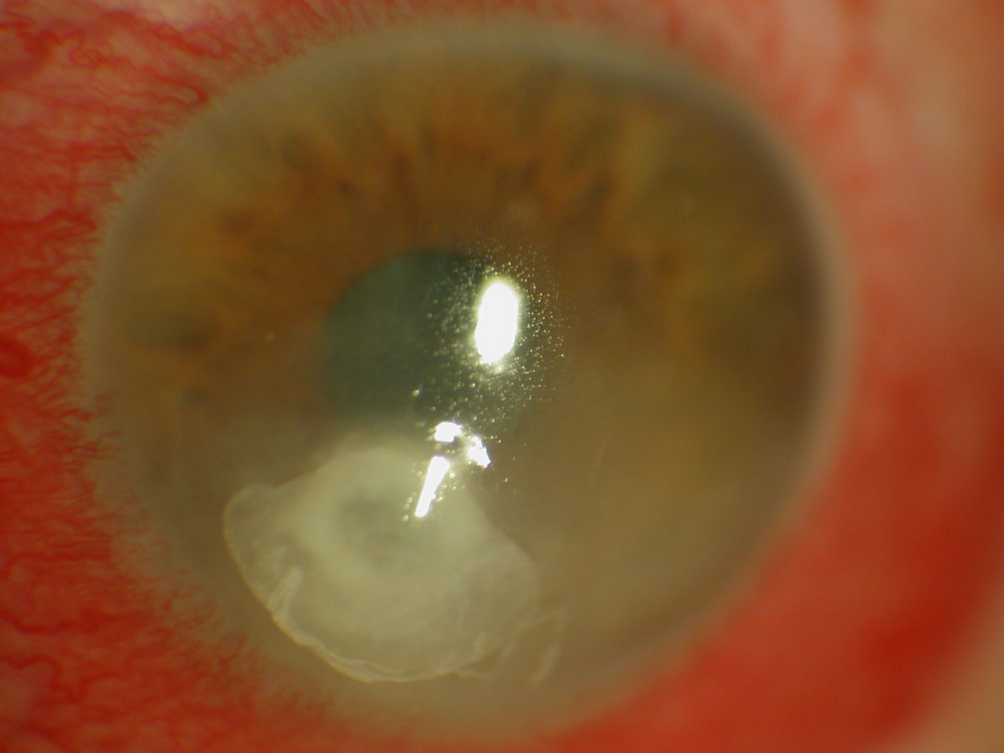
Corneal ulcer and infiltrates in a case Candida keratitis; the signs are similar to those found in bacterial keratitis

Signs and symptoms of FK and are summarised in Table 1. The signs vary with whether the fungi are filamentous or yeast (*Candida species*).^100^, ^101^, ^104^, ^105^ Figure 5 illustrates the classic signs of Candida keratitis with a stromal infiltrate, overlying epithelial defect, and conjunctival hyperaemia; similar to BK (Figures 1 and 2). Whereas in filamentous FK the stromal infiltrate may have feathery margins and there maybe satellite lesions with a thick endothelial exudate.

#### 3.3.3 Diagnostic methods

##### Microbiology

Staining and corneal scrape culture are the preferred diagnostic methods for FK.^102^, ^104^ Direct microscopy is a very valuable and fast method to detect fungal filaments from corneal scrapes. About 65% to 75% of Gram or Giemsa are positive to fungal hyphae.^14^, ^101^ The 10% potassium hydroxide (KOH) staining is another common procedure with sensitivity between 61% and 99.23% and specificity between 91% and 97%.^100^, ^101^

Culture media such as blood and chocolate agar and Sabouraud dextrose agar have been used to isolate and identify fungi.^99^ Sabouraud agar has a lower pH and sometimes with addition of antibiotics, this agar can be tailored to selectively grow fungi instead of bacteria.^104^ Fungal culture media should be maintained at 22 to 25 degrees in a cooling incubator if available. Fungi can be confirmed in blood agar and Sabouraud agar at a minimum of 48 to 72 hours.^99^ Brain‐heart infusion and thioglycolate broth liquid media can also be used; but there are not selective for fungi.^104^ *C. albicans* appear as smooth, glossy, raised, cream‐coloured colonies clustered together on Sabouraud dextrose agar; while *Fusarium species* grow as flat and spreading colonies with feathery borders. Despite being the gold standard for diagnosis, the culture sensitivity from corneal scrapings is limited with low rates of 25%, as fungi can take days or weeks to grow.^99^ In vitro susceptibility tests for fungi are not performed routinely due to poor correlation to the clinical response.^106^ Corneal biopsies may be needed to isolate the causal organism, as filamentous fungi grow slowly in culture, and for progressive infections despite an adequate antimicrobial therapy.^98^

##### In vivo confocal microscopy

Another non‐invasive imaging tool is in vivo confocal microscopy (IVCM) which provides in vivo images of the cornea with a resolution of 1 μm, from the epithelium to endothelium, nerves and cells, sufficient to yield images larger than a few micrometres of filamentous fungi or Acanthamoeaba cysts.^97^, ^99^, ^103^ Sensitivity of IVCM has been reported as between 80% and 94%, and specificity between 78% and 91.1%.^98^, ^99^ It has been reported to be of variable value in the diagnosis and monitoring of fungal and acanthamoeba keratitis (AK)^106^ and is highly dependent on the experience of the observer.^107^, ^108^

Advantages of IVCM include ‘non‐invasiveness’, real‐time and early identification of the organism, for monitoring and guidance of the therapy, and determination of the depth of the infection. Limitations of IVCM include the need for an experienced operator, patient co‐operation, unsuitability for smaller organisms, motion artefacts and dense corneal infiltrates and/or scarring can affect the proper tissue penetration and visualisation.^99^, ^100^, ^104^ Typically, IVCM is performed in cases of progressive keratitis and/or when acanthamoeba or FK are suspected. Anterior segment OCT has also been used to image the cornea, it is emerging as a diagnostic tool in microbial keratitis.^110^

##### Polymerase chain reaction test

The sensitivity of the PCR ranges from 75% to 100%, and specificity from 50% to 100% for the diagnosis of FK compared to the corneal scrape culture.^29^, ^97^, ^99^, ^103^ In culture or staining negative results, the PCR has the highest positive detection rate. PCR advantages include that a small sample is required for diagnosis, yielding a fast result within 4 to 8 h when available, compared to cultures results which are available between 2 to 7 days. PCR also appears to be useful in earlier infections with low fungal load.^99^, ^103^ Major disadvantages are its high cost and lack of wide availability. Nevertheless, PCR is a supplementary diagnostic tool to guide early antifungal therapy while awaiting for other diagnostic test results.^99^

#### 3.3.4 Management

Management of FK includes antifungal agents, cycloplegics to relieve anterior uveitis, antibiotics for secondary bacterial infection if present and surgical intervention if required.^98^ FK generally has poor clinical outcomes due to the reduced ocular penetration and efficacy of antifungal medications and the difficult diagnosis of this condition to commence an adequate initial therapy.^98^ Management of FK includes antifungal agents, cycloplegics to relieve anterior uveitis, antibiotics for secondary bacterial infection if present and surgical intervention if required.^98^

The selection of antifungal medications may depend on their availability, clinician preference and consultation with infectious diseases specialists.^98^ Topical natamycin 5% is FDA approved and commercially available in the United States and has been associated with better outcomes in *Fusarium* keratitis, despite its poor penetration. Topical voriconazole and amphotericin B 0.15% can also be considered as alternatives. Topical voriconazole's limitations include its cost and being less effective than topical natamycin. Topical amphotericin can be prescribed as first choice to yeasts and as alternative to filamentous fungi; its limitations include its preparation and stability.^97^, ^98^, ^100^, ^104^

Oral medications such as voriconazole, ketoconazole, itraconazole or oral fluconazole may be added; although the Mycotic Ulcer Treatment Trial 2 (MUTT 2) concluded that oral voriconazole made no difference in the treatment of severe filamentous keratitis and the incidence of corneal perforation.^104^, ^111^ Posaconazole is a new medication; its mechanism of action is blocking fungal cell wall ergosterol synthesis. It has a broad‐spectrum activity against *Candida species, Aspergillus species* and *Cryptococcus neoformans*. It is also effective in cases of *Fusarium species* resistant to other antifungals without toxicity.^100^ Intracameral or intrastromal antifungals maybe considered when the infection involves the deep stromal layers, significant anterior chamber reaction and ulcers not responding topical and oral medication as well as during corneal transplant surgery for FK.^100^, ^104^, ^112^ The risk of corneal scarring from intrastromal injection must be weighed against that of progressive infection. A PK is indicated when the medical therapy has failed and maybe considered earlier in progressive keratitis, severe corneal thinning, impending perforation and keratitis involving the limbus. Unfortunately, PK has a high rate of recurrent infections ranging from 5% to 14%, usually in cases which involve the limbus and with preoperative hypopyon and corneal perforation. In addition, a study from India reported the media graft survival of 5.9 months with two risk factors: size of corneal infiltrate and size of corneal graft.^100^, ^104^

### 3.4 Microsporidial keratitis

Microsporidia are unicellular organisms from the phylum Microspora and kingdom Protista. They have been reclassified as fungi. The intracellular spore is the infectious form of the organism.^113^, ^114^ The infection can be transmitted via faeco‐oral, contaminated water or food for intestinal microsporidosis; however, the source for ocular infections is unknown.^33^, ^114^ Risk factors for this infection include CLW, rainy season and exposure to muddy water.^33^

#### 3.4.1 Clinical features

This organism can cause keratoconjunctivitis; usually in immunocompromised patients; endophthalmitis and stromal keratitis, in immunocompetent patients.^33^, ^113^ The infection is typically insidious, difficult to diagnose, and often mistaken for viral keratitis.^33^, ^113^ It can present as epithelial keratopathy or stromal keratitis, which is less common than keratoconjunctivitis. Stromal keratitis presents with diffuse congestion, greyish white stromal infiltration, oedema without suppuration, or deep stromal infiltrate with or without an overlying epithelial defect.^33^, ^114^

#### 3.4.2 Diagnostic tests

This organism can be identified as bright turquoise to white intracellular oval bodies clustered in groups against a dark background in 0.1% calcofluor white or 10% potassium hydroxide (KOH) stains.^33^, ^113^, ^114^ Bright purple, ovoid, refractile spores similar to Gram‐positive organisms can be seen in Gram stains. Calcoflour white and modified Ziehl‐Neelsen stains are the most sensitives stains for identifying this organism.^33^, ^114^ Madin‐Darby canine kidney (MDCK), Vero, HeLa and SIRC cell lines culture media can be used to grow Microsporidia. Other tests such as PCR and transmission electron microscopy (TEM) can be used to identify the species.^33^ TEM is the gold standard for diagnosis of microsporidial spores but it is not easily accessible to most laboratories and further tests are needed to determine the species.^114^ Pan microsporidian 16S rRNA has been used to identify the microsporidial species with a sensitivity of 83% and specificity of 98%.^114^ A microsporidial infection should be considered as a differential diagnosis in culture‐negative stromal keratitis not responding to standard antimicrobial therapy.^33^, ^114^

#### 3.4.3 Treatment

There is no standard therapy for microsporidal infection. Therapies with albendazole, itraconazole propamidine isethionate 0.1%, PHMB 0.02%, chlorhexidine 0.02%, voriconazole 1%, fluconazole 0.3% and fumagillin 0.3% have had some success requiring a long‐term therapy for several weeks.^33^, ^114^ Fluoroquinolones have also been used in combination with albendazole and topical fumagillin.^33^ Therapeutic penetrating keratoplasty may be needed in a non‐responding infection to medical therapy and for definitive therapy.^33^ Microsporidia stromal keratitis has poor clinical outcomes and surgery is needed in most of the cases.^33^, ^113^, ^114^

### 3.5 *Acanthamoeba* keratitis

*Acanthamoeba species* are ubiquitous free‐living amoebae. At least 24 amoebic protozoa species exist worldwide, and they exist in both soil and nearly all water sources. Human ocular involvement with *Acanthamoeba* presents in the form of keratitis. AK is a rare, sight‐threatening infection. The incidence of AK differs between developed and developing countries, as well as between geographical areas.^115^

#### 3.5.1 Predisposing factors

The incidence of AK is lower in developing countries compared with developed countries.^116^ In the latter, the majority of cases are linked with CLW, specifically soft CLs.^116^, ^117^ Diagnosis is often late due to its low incidence of around 3%–15% in the United Kingdom and United States^115^ and 3.6 cases per year in Australia.^118^ In developing countries like India, CLW is less prevalent and most AK cases are associated with trauma.^119^ In non‐CLW, AK cases are associated with contaminated soil, water and surgical trauma. Younger age is associated with increased incidence of AK, this may be related to the increased prevalence of CLW worldwide.^120^, ^121^ The infection is often caused by contamination during cleaning procedures.^115^ Furthermore, warmer periods of the year (i.e., summer) are associated with higher incidence. This is because during the warmer months there is an increased number of amoebae in surface water and prolonged water activities occur.^122^, ^123^

#### 3.5.2 Clinical features

**FIGURE 6:**
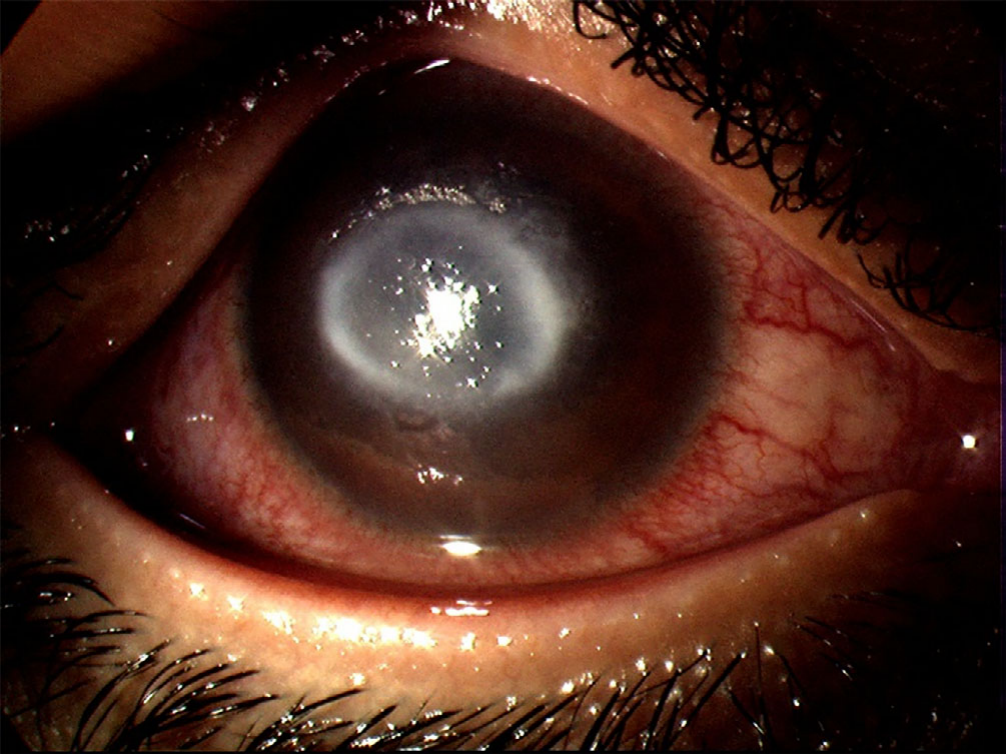
Ring infiltrate in acanthamoeba keratitis

**FIGURE 7:**
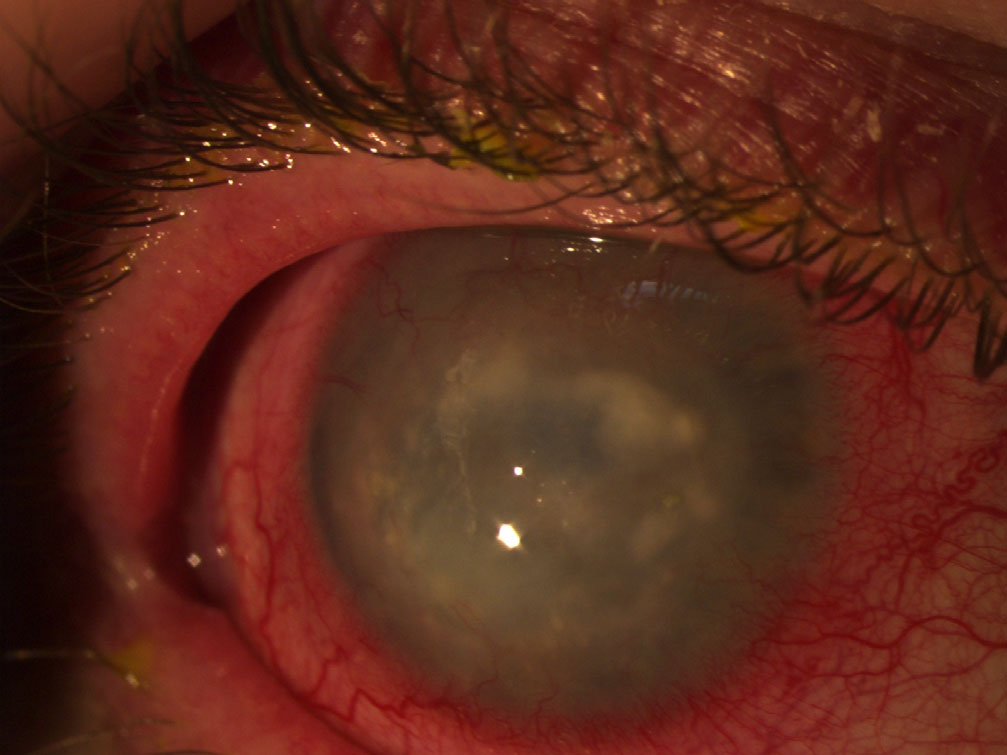
Advanced acanthamoeba keratitis, scattered stromal infiltrates with corneal vascularisation and conjunctival hyperaemia are noted

Symptoms and signs are described in Table 1 and in Figures 6 and 7. During the early stages, patients may also present with eyelid ptosis, conjunctival hyphemia and pseudodendrites. Keratoneuritis or radial nerve enlargement with perineural infiltrates maybe present but are not pathognomonic, as they may also occur in pseudomonas keratitis and be absent late in the disease.^124^, ^125^ Deep stromal infiltrates, corneal perforation, satellite lesions, scleritis and anterior uveitis with hypopyon may occur as the disease progresses.^118^

#### 3.5.3 Diagnostic tests

**FIGURE 8:**
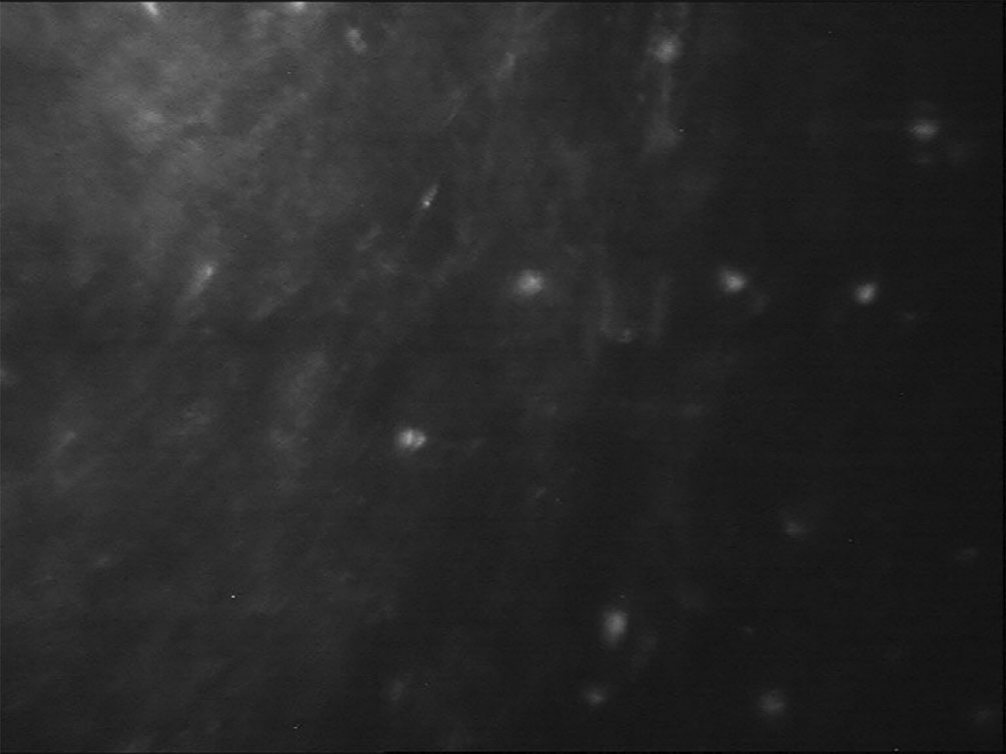
In vivo confocal microscopy of acanthamoeba keratitis

A provisional diagnosis of AK can be made from the patient's history, clinical features and IVCM (Figure 8). During IVCM, *acanthamoeba* cysts appear as hyperreflective, spherical and well‐defined double‐wall structures, while trophozoites are difficult to discriminate from leukocytes and keratocyte nuclei.^126^ Identification of *Acanthamoeba species* via corneal scrape or PCR should also be performed to confirm diagnosis. Epithelial debridement as part of the scrape procedure can also assist management by reducing the acanthamoeba load. The culture specimen should then be inoculated onto *Escherichia coli* plated over non‐nutrient agar. Cultures for bacterial, fungal and viral infections should also be performed as early clinical signs are nonspecific and indistinguishable from other types of keratitis.^127^ While, culture on *E. coli* agar plates remains the gold standard for diagnosing A*canthamoeba species*, PCR testing has become well established and demonstrated to have higher sensitivity than corneal culture (67% to 75% vs. 31% to 33%).^128^, ^129^ Furthermore, *E. coli* plates may not be available in all centres.^128^, ^130^ In the case of deep corneal involvement, a corneal biopsy may be needed for diagnosis.^27^

#### 3.5.4 Treatment

AK is a complicated infection, however, early diagnosis and aggressive medical therapies have improved the management of this disease. A combination of various topical acanthamoeba agents is usually utilised as no single drug can eliminate both cystic and trophozoite forms. The cyst form tends to be highly resistant to therapy, therefore, a combination of agents is generally used. Polyhexamethylene biguanide (PHMB) and chlorhexidine are topical agents effective against acanthamoeba trophozoites, with variable efficacy against cysts.^121^, ^130^ Chlorhexidine is often used in combination with propamidine or hexamidine and has shown good results if the treatment is commenced early during the course of infection.^131^ However, propamidine and hexamidine are not available in all countries.

Post corneal scrape procedure, topical anti‐acanthamoeba drugs should be administered every hour for the first several days, the frequency then reduced depending on clinical response. Treatment is recommended for 6 to 12 months with close observation to prevent recurrent infection.^132^ Therapeutic penetrating keratoplasty is reserved as a measure of last resort in cases of impending corneal perforation. Robaei et al. suggest delaying corneal transplantation where possible until the eye is no longer inflamed and after completion of anti‐acanthamoeba treatment.^133^ Penetrating keratoplasty should be considered when the infection spreads to the paracentral corneal stroma, as performing this procedure on a more localised infection may allow for the total removal of the organism.^134^ To control inflammation, topical steroids may be used but only after anti‐acanthamoeba therapy has been commenced.^134^, ^135^ To control inflammation, topical steroids may be used but only after anti‐acanthamoeba therapy has been commenced.^135^

Complications such as scleritis and treatment toxicity can occur. Clinicians should instruct patients on proper cleaning of CLs and remind patients to avoid wearing CLs while swimming or showering^136^ as this can prevent the occurrence of the disease.

## 4 CONCLUSION

Infectious keratitis is the fifth leading cause of blindness overall worldwide. Early diagnosis and adequate therapy are key to avoid complications such as vision impairment and blindness. For bacterial, fungal and AK, culture of corneal scrapes is the initial diagnostic test to grow and identify the causing organism. Alternative diagnostic tools such as PCR and IVCM can be also used to aid determination of the causal organism(s). In HSK, the diagnosis is mainly based on clinical examination. PCR testing can also be used; however, it is not useful in stromal and endothelial HSK due to their immune‐related pathogenesis. Newer diagnostic tests such as NGS and deep learning models are being used in selective health settings with the hope that they maybe widely utilised in the near future. Challenges remain in infectious keratitis. First, educating patients with predisposing factors such CLW, OSD or agricultural workers about the risks of infection is crucial to avoid acquiring the infection and encouraging early presentation. Second, developing new diagnostic tests to determine the causal organism in a timely manner, with good sensitivity and specificity while being cost‐effective. Finally, a judicious use of antimicrobials is needed to avoid increasing AMR rates which may lead to sight‐threating complications.

## FUNDING INFORMATION

This study was funded by the Sydney Eye Hospital Foundation.

## CONFLICT OF INTEREST

The authors declare no conflicts of interest.
